# Collecting Real-World Data via an In-Home Smart Medication Dispenser: Longitudinal Observational Study of Survey Panel Persistency, Response Rates, and Psychometric Properties

**DOI:** 10.2196/60438

**Published:** 2025-02-03

**Authors:** Benjamin Ogorek, Thomas Rhoads, Erica Smith

**Affiliations:** 1 Spencer Health Solutions Inc, 2501 Aerial Center Pkwy, Suite 100, Morrisville, NC, 27560, United States, 1-866-971-8564; ^2^WCG, Princeton, NJ, United States

**Keywords:** real-world data, real-world evidence, patient-reported outcomes, longitudinal studies, survey methods

## Abstract

**Background:**

A smart medication dispenser called “spencer” is a novel generator of longitudinal survey data. The patients dispensing medication act as a survey panel and respond to questions about quality of life and patient-reported outcomes.

**Objectives:**

Our goal was to evaluate panel persistency, survey response rates, reliability, and validity of surveys administered via spencer to 4138 polychronic patients residing in the United States and Canada.

**Methods:**

Patients in a Canadian health care provider’s program were included if they were dispensing via spencer in the June 2021 to February 2024 time frame and consented to have their data used for research. Panel persistency was estimated via discrete survival methods for 2 years and survey response rates were computed for 1 year. Patients were grouped by mean response rates in the 12th month (<90% vs ≥90%) to observe differential response rate trends. For reliability and validity, we used a spencer question about recent falls with ternary responses value-coded −1, 0, and 1. For reliability, we computed Pearson correlation between mean scores over 2 years of survey responses, and transitions between mean score intervals of [0, 0.5), [−0.5, 0.5), and [0.5, 1]. For validity, we measured the association between the falls question and known factors influencing fall risk: age, biological sex, quality of life, physical and emotional health, and use of selective serotonin reuptake inhibitors or serotonin-norepinephrine reuptake inhibitors, using repeated-measures regression for covariates and Kendall τ for concomitant spencer questions.

**Results:**

From 4138 patients, dispenser persistency was 68.3% (95% CI 66.8%‐69.8%) at 1 year and 51% (95% CI 49%‐53%) at 2 years. Within the cohort observed beyond 1 year, 82.3% (1508/1832) kept surveys enabled through the 12th month with a mean response rate of 84.1% (SD 26.4%). The large SD was apparent in the subgroup analysis, where a responder versus nonresponder dichotomy was observed. For 234 patients with ≥5 fall risk responses in each of the first 2 years, the Pearson correlation estimate between yearly mean scores was 0.723 (95% CI 0.630‐0.798). For mean score intervals [0, 0.5), [−0.5, 0.5), and [0.5, 1], self-transitions were the most common, with 59.8% (140/234) of patients starting and staying in [0.5, 1]. Fall risk responses were not significantly associated with sex (*P*=.66) or age (*P*=.76) but significantly related to selective serotonin reuptake inhibitor or serotonin-norepinephrine reuptake inhibitor usage, quality of life, depressive symptoms, physical health, disability, and trips to the emergency room (*P*<.001).

**Conclusions:**

A smart medication dispenser, spencer, generated years of longitudinal survey data from patients in their homes. Panel attrition was low, and patients continued to respond at high rates. A fall risk measure derived from the survey data showed evidence of reliability and validity. An alternative to web-based panels, spencer is a promising tool for generating patient real-world data.

## Introduction

### Background

The use of patient data collected in real-world settings has never been more impactful. The US Food and Drug Administration’s Real-World Evidence (RWE) Program has elevated real-world data (RWD) as a tool to support new indications for already approved drugs [1-3], the European Medicines Agency has published their RWE framework [4], and Canada’s Drug and Health Technology Agency has published their guidance document [5].

RWD may take the form of claims records, electronic health records, registries, or patient-generated data, with patient-reported outcomes (PROs) as an important subset. Longitudinal surveys, where patients are surveyed at 2 or more points in time, generate data that allow for the analysis of within-unit change as well as aggregations over time [6]. This results in greater “causal leverage” than cross-sectional surveys [7] and is ideal for submissions to regulatory bodies.

Web-based panels, or “registered persons who have agreed to take part in online studies on a regular basis,” rose in attractiveness with the proliferation of the web [8]. Recently, however, shortcomings of longitudinal studies based on web-based panels have undermined their reputation as a high-quality data source. Panel attrition, where subjects in earlier waves cease to respond in later waves, has become worse since the 1990s [7,9-11]. While web-based panel data are also prone to quality problems (eg, false answers, careless responses, and multiple panel memberships [12]), these problems have been exacerbated by innovations in automation and improvements in large language models, where human reviewers are unable to consistently detect automated responses [13]. This has become a corrupting force in web-based survey sampling [14].

Amazon Mechanical Turk (MTurk) was considered a representative and convenient source of web-based longitudinal survey data [15] but has seen its reputation deteriorate within the last decade. For example, a study that used MTurk to build a diabetes panel failed after only 5.8% (13/224) were deemed eligible for future survey research [16]. Researchers noted declines in MTurk data quality starting around summer 2018, as evidenced by degraded psychometric properties of well-understood metrics [17]. A warning was issued in the journal *Perspectives on Psychological Science* after an exercise revealed that only 2.6% (14/529) of MTurk samples were valid [18].

**Figure 1.**
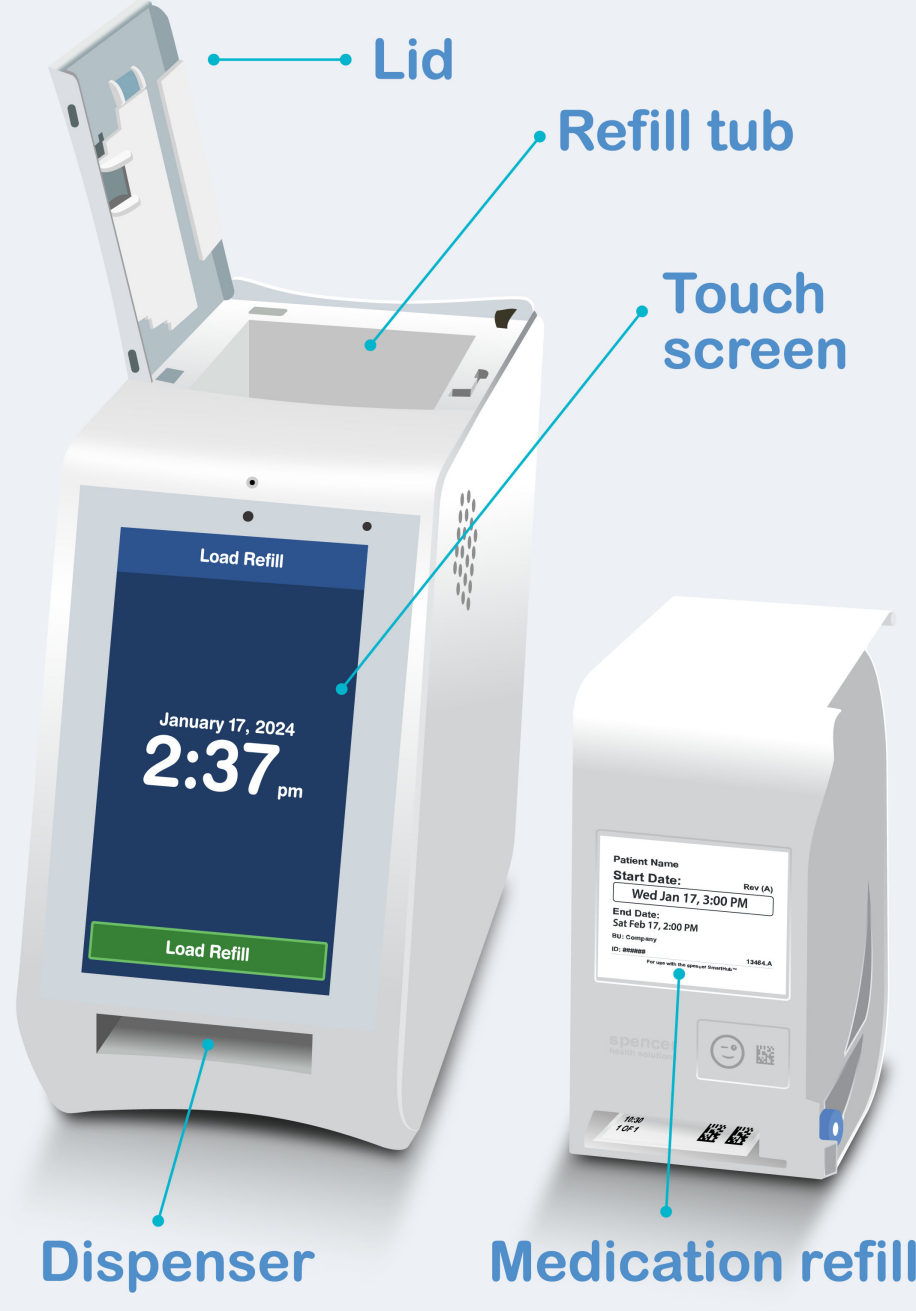
Key components of the spencer smart medication dispenser.

Alternatives to web-based panels exist in populations of patients using web-connected hardware, also known as “smart” products. One interesting subset is the population of patients using smart medication dispensers, as these products sit in the home amidst a public health need for digital adherence solutions [19]. A 2023 review of smart medication adherence products reviewed the features of 51 products without mention of survey administration capabilities [20]. One of these products, a dispenser named “spencer” [21,22] (manufactured by Spencer Health Solutions, Inc), has a touch screen display that allows it to administer survey questions following on-time medication dispenses (Figure 1).

At the time of writing, in-home spencer devices have generated more than 3 million longitudinal responses from more than 4000 unique patients to quality of life and PRO measures from a polychronic population residing in the United States and Canada. These are patients of Canadian health care provider Custom Health, Inc, a company offering “a personalized, connected service that goes beyond medication management and ensures medications are working as they should” [23]. Patients or caregivers can express interest directly via a collection of sign-up forms [24,25] or they may be directed to spencer via their health plans that have partnered with Custom Health [26]. When a health plan partners with Custom Health, their services are provided to members “who require a high degree of clinical oversight, those managing multiple medications or those experiencing medication adherence challenges” [27].

### Objectives

The study’s aim was to evaluate the spencer smart medication dispenser as a longitudinal survey platform for a polychronic patient population. Panel persistency, survey response rates, and measurement reliability and validity were assessed.

## Methods

### Recruitment

Patients of Custom Health were included if they met the criteria enumerated in Textbox 1.

Textbox 1. **Inclusion criteria for patients in the study.**

1. The patient entered Custom Health’s intake process either by self-selection or based on the recommendation of a health care provider.
2. Custom Health professionals decided to pair the patient with a spencer smart medication dispenser.
3. The patient agreed to the Custom Health consent form.
4. The patient agreed to the Spencer Health Solutions End User License Agreement, permitting his or her deidentified data to be used for research purposes. This occurred on the spencer unit’s touch screen.
5. The patient’s first scheduled medication dose was between June 3, 2021, and February 14, 2024.
6. The patient dispensed a medication dose by March 14, 2024. In this paper, dispensing medication refers to dispensing multidose packs containing oral solids.

After completing Custom Health’s intake process, spencer devices were shipped to patients’ homes. Once set up, the devices displayed both current local time and the scheduled time of the next medication dispense via a touch screen display. Refills containing medication strips (multidose adherence packaging) prepared by a pharmacy were shipped to the patients’ homes and were inserted by the patient or care nurse into the top of the unit via an electronically controlled door. At scheduled dosing times, the unit alerted the patient through sound, light, and a message on the touch screen display. After the patient pressed the dispense button on the touch screen, the unit dispensed 1 or more medication pouches. After an on-time dispense, a question was presented to those patients who had not explicitly opted out of surveys.

### Data Generation and Processing

The question and response mechanism is further elaborated here. If a dose was dispensed on time and the patient had not opted out of surveys, 1 question was displayed on-screen. To answer, a single button press was needed to select from a multiple-choice answer set. This was followed by a review step (also serving as the completeness check) where the patient could confirm the selection or go back and change the answer. If a patient did not make the confirmation in the review step, the questionnaire would not be submitted to the database and later analyzed as an instance of nonresponse. If left attended to, a question would remain on the screen until the next scheduled dose.

In collaboration with health care professionals at Custom Health, 35 survey items were designed to measure the spencer experience, quality of life, and PROs. To avoid copyright infringement, these questions were not taken from any existing validated scale. Questions were scheduled one-to-one with doses in a predefined sequence that repeated indefinitely. Response options were consistently listed from most positive sentiment (eg, “Excellent”) toward the top of the screen to least positive sentiment (eg, “Poor”) toward the bottom of the screen. Questions were manually answered on test devices in a quality assurance laboratory before being released to patients, and patients could call into a support line to provide feedback regarding the questions or to request that they be turned off.

As is typical of web-based panels, the panel formed by the selection criteria in Textbox 1 constitutes a convenience sample. The target population best described by the sample is polychronic patients taking multiple medications daily. Since the surveys were administered as part of routine patient monitoring, no institutional review board (IRB) approval was needed.

Survey responses were sent to the application database through either cellular connection (the default) or Wi-Fi. In cases where the spencer lost connectivity, a store and forward mechanism sent data to the cloud database once connectivity was reestablished. The database is managed by Spencer Health Solutions that has received both ISO27001 [28] and Data Privacy Framework [29] certifications.

Date of birth, biological sex, and residential postal code were entered into a web-based portal by health care providers when patients were recruited. These fields were retrieved from the application database March 4, 2024. Dates of birth that were within 2 years of the database entry date were replaced with missing values, and age was computed as the difference between the first dispense date and date of birth. Within the United States, 5-digit postal codes were converted into US states via the *zipcodeR* R package. For Canadian postal codes, a function was written that maps the first letter of the postal code to the associated province. Prescription information was created by the pharmacies at the time of refill creation and sent to the database.

### Statistical Analysis

#### Panel Attrition

Patients may leave the spencer dispensing platform for multiple reasons, including life transitions to higher care services or natural death. Leaving the dispensing platform is the primary mechanism of spencer panel attrition. To estimate dispenser persistency, we used the discrete survival analysis framework described by Allison [30], where the periods start on the first day a patient is scheduled, are 30 days in length, and an attrition event occurs when a patient is not scheduled during an entire 30-day period. There is a *resurrection* mechanism: when a patient is scheduled in a later period after previously meeting the definition for an attrition event, the attrition flag is reset for all previous periods.

For readability, we will refer to a 30-day period as a *month*, 12 thirty-day periods as a *year*, and so on. Furthermore, we will refer to the time in years between the first scheduled dose via spencer and the analysis date as *tenure*. A patient’s tenure represents the amount of experience a patient has had with the spencer platform as of an analysis date.

Beyond dispenser attrition, the second source of panel attrition is when patients request that their questions no longer appear on-screen following a dispensing event. To study this phenomenon, we computed rates of requested question discontinuation for the first 12 months of tenure for patients who remained dispenser persistent for more than a year.

When pursuing the subset of patients who were dispenser persistent for more than a year, the subset taken was patients who remained on the spencer platform through the 14th month. In addition to our operational month being shorter on average than a calendar month (by a fraction of a day), the 2 additional months of persistency provided a buffer against the decreased interaction with the device that often precedes full platform discontinuation.

#### Survey Response Rates

On the survey platform, nonresponse occurs when patients do not enter a response after a question is displayed and the question is cleared. We computed rates of nonresponse by month and plotted the resulting series. We knew from prior analyses that some patients consistently respond to the questions, and we wanted to observe this phenomenon. For patients who were still receiving surveys in the 12th month, we created 2 groups: those with 12th-month response rates of <90% and those with 12th-month response rates of ≥90%. For both groups, we computed the frequency of patients, plotted response rates by month, and provided a qualitative description of the patterns observed.

### Psychometric Analysis

#### Reliability

A reliability analysis in the context of a platform requires a narrowing of focus to a specific measure, as both reliable and unreliable measures may be generated from any platform. Inspired by the Falls Efficacy Scale-International, a reliable measure of fear of falling known to be related to both past and future falls [31,32], we chose an existing question from our rotation that asks the patient about recent falls. Hereafter referred to as Q_FALL, the question text read “Have you experienced a fall in the past month?” The response options were “No,” “Not Sure,” and “Yes” (a 1-letter variation in capitalization occurring after September 2022, where “Not sure” was replaced with “Not Sure”), which were value-coded as 1, 0, and −1, respectively.

One conceptualization of reliability is test-retest reliability and can be quantified using Pearson correlation between a measure’s values at 2 time points [33]. For a comparative baseline in the literature, Falls Efficacy Scale-International measurements taken by the same patients at different time intervals had Pearson correlations ranging from 0.66 to 0.83 for measurements taken up to a year apart [34].

The Pearson correlation coefficient is known to suffer bias when distributional assumptions are violated, a concern because Q_FALL has only 3 response levels and there were different response counts between patients and years. The use of averages and bootstrap resampling were thus employed to address these factors. First, we limited attention to a subset of 234 patients from the persistency analysis who answered Q_FALL at least 5 times in both a full first year and a second year of tenure, hereafter referred to as year 1 and year 2. Second, we used the bootstrap to obtain a bias-corrected estimate of the Pearson correlation along with a nonparametric 95% CI [35]. This allowed us to perform inference on the coefficient of determination (*R*^2^) for the equivalent regression of the year 2 means regressed on the year 1 means.

Averaging the ternary scores allowed us to work on a continuum where rare fallers and never fallers appear close together on the resulting scale, a notion supported by similarities between these groups in a 1-year cohort study [36]. To circumvent the limitations of a linear correlation analysis, we performed an additional discrete state transition analysis. We examined the frequency of transitions to and from mean Q_FALL scores of [−1, −0.5), [−0.5, 0.5), and [0.5, 1.0] in year 1 and year 2, expecting self-transitions to be the most frequent.

#### Validity

To assess the convergent validity of the recent falls question administered via spencer, the mean scores for year 1 and year 2 were compared with the following established risk factors of fall risk: increased age, biological sex, previous fall frequency, low quality of life, depressive symptoms, physical impairment, and medication use [31,34,37]. Many patients in this population were prescribed selective serotonin reuptake inhibitors (SSRIs) or serotonin-norepinephrine reuptake inhibitors (SNRIs), and these are associated with falls in the older adults [38,39]. A meta-analysis found that 95% (70/74) of studies reported gender or sex differences in fall-related outcomes with females at a higher risk than males [40]. Canonically, increased age is a risk factor for falls [41]. The validity analysis was split into 2 parts, each based on the 234-patient subset from the reliability analysis.

We first conducted an analysis of the relationship between raw Q_FALL values and covariates age, sex, and SSRI or SNRI usage, as these were known before any responses were received (medication can be discontinued but medication classes tend to be stable within patient). To accommodate the repeated measures received from each patient, we used a generalized estimating equation approach to model the relationship between the coded value of Q_FALL and an exchangeable working correlation structure. This was accomplished with the *geepack* package in R, which reports SEs that are robust to both the choice of working correlation structure and nonnormality of the response. For age and SSRI or SNRI usage, we expected to see negative relationships. For biological sex, we expected that female patients would be associated with lower mean Q_FALL than male patients.

For evidence of association between Q_FALL and other relevant variables, including quality of life, depressive symptoms, and hospital visits, we selected the questions listed in Table 1 as contemporaneous survey-based measures that had face validity for concepts of interest. Their responses are integer-coded and arranged by sentiment, and thus we expected positive correlations with Q_FALL.

The robust Kendall τ measure was used to test for associations, as the sample sizes of the questions from Table 1 may be arbitrarily small within patient. Kendall τ is more appropriate for ties and has an accompanying 2-sided nonparametric test for testing the null hypothesis of zero association [35]. For a nonparametric 95% CI on τ, we used the *kendall.ci* function from the R package *NSM3* [42], which provides a bootstrap CI.

**Table 1.** Standard spencer questions relating to known risk factors of falling.

Question text	Possible responses	Values coded	Construct^a^
Rate your recent quality of life.	Excellent | Very good | Good | Fair | Poor	5 | 4 | 3 | 2 | 1	Quality of life
How is your emotional health today?^b^	Excellent | Very good | Good | Fair | Poor	5 | 4 | 3 | 2 | 1	Depression
How would you rate your physical health today?	Excellent | Very good | Good | Fair | Poor	5 | 4 | 3 | 2 | 1	Physical health
Rate your ability to perform activities today.	Excellent | Very good | Good | Fair | Poor	5 | 4 | 3 | 2 | 1	Ability or disability
Are you able to accomplish what you have planned today?	Completely | Mostly | Moderately | A little | Not at all	5 | 4 | 3 | 2 | 1	Ability or disability
Hospital, ER^c^, or urgent care in the past month?	No | Not sure | Yes	1 | 0 | −1	ER visits from falls

a Construct is based on face validity of the spencer standard questions.

b This question has been in rotation for multiple years, but in September 2022, the number of responses changed from 3 (“Poor,” “Good,” and “Excellent”) to 5 (“Poor,” “Fair,” “Good,” “Very good,” and “Excellent”). We coded the 3-response set as 1, 3, and 5, and the 5-response set as 1, 2, 3, 4, and 5, respectively.

c ER: emergency room.

### Ethical Considerations

This study used operational data collected from a commercial medication dispensing system used in routine patient care and was not subject to IRB review requirements, so IRB approval was not pursued. Users of the spencer device provided consent for data collection through the End User License Agreement, which covers the collection of medication adherence data and responses to quality of life and PRO surveys as part of the system’s standard operation. No additional compensation was provided to users beyond the normal terms of their device usage agreement. All data analyzed in this study were deidentified prior to analysis. Spencer Health Solutions has achieved both ISO27001 [28] and Data Privacy Framework [29] certifications, and the system uses industry-standard encryption and security measures.

This research analyzed data collected during standard clinical care and device usage. All results are presented as anonymous aggregate statistics. The original data collection occurred as part of routine clinical practice, with patients providing consent for research use through the device terms of service and care management agreement. Under Canadian TCPS 2 Article 2.4, research ethics board review is not required for research that relies exclusively on secondary use of anonymous information where the process does not generate identifiable information. Under US regulation 45 CFR 46.104(d)(4)(ii), IRB review is not required when information is recorded by the investigator in such a manner that subjects cannot be identified, directly or through identifiers linked to the subjects, the investigator does not contact the subjects, and the investigator will not reidentify subjects.

## Results

### Patient Population

The patient population was majority female (2552/4133, 61.7%), with 0.1% (5/4138) of the biological sex fields missing. The mean age was 54.4 years (SD 19.9, range 5-104 years). Most patients (3736/4138, 90.3%) resided in Canada, with 1 address unmapped at the country level. Patients were scheduled to take multiple drugs per day (mean 9.6, SD 5.1). Of the 2805 unique compounds scheduled during the observation window, 70.2% (1970/2805) were mapped to an Anatomical Therapeutic Chemical classification system second-level code, with a modified “Vitamins & Supplements” that included dietary supplements. Table 2 contains the 20 most frequently observed subgroups observed during the observation window.

**Table 2.** Patient demographics, geographic distribution, and medication usage among the 4138 patients studied.

Section and variable	Patients (N=4138)
Patient demographics, n (%)	
Sex	
Female	2552 (61.7)
Male	1581 (38.2)
Missing	5 (0.1)
Age (years)	
Valid records, n (%)	4123 (99.6)
Invalid records, n (%)	15 (0.4)
Mean (SD)	54.4 (19.9)
IQR	39‐70
Age range	5‐104
Geographic distribution, n (%)	
Country	
Canada	3736 (90.3)
United States	401 (9.7)
Missing or other	1 (0)
Canadian provinces	
Ontario	2083 (50.3)
British Columbia	1149 (27.8)
Saskatchewan	485 (11.7)
Other	19 (0.5)
US states	
Tennessee	221 (5.3)
Missouri	117 (2.8)
California	32 (0.8)
Ohio	24 (0.6)
Other	7 (0.2)
Medication usage (Anatomic Therapeutic Chemical codes, second level), n (%)	
Psychoanaleptics	3040 (73.5)
Vitamins & supplements	1917 (46.3)
Lipid-modifying agents	1848 (44.7)
Drugs for acid-related disorders	1788 (43.2)
Antiepileptics	1717 (41.5)
Agents acting on the renin-angiotensin system	1664 (40.2)
Psycholeptics	1503 (36.3)
Drugs used in diabetes	1317 (31.8)
Beta-blocking agents	1107 (26.8)
Antithrombotic agents	1050 (25.4)
Calcium channel blockers	893 (21.6)
Diuretics	822 (19.9)
Thyroid therapy	741 (17.9)
Urologicals	592 (14.3)
Analgesics	543 (13.1)
Anti-inflammatory and antirheumatic products	378 (9.1)
Antihypertensives	345 (8.3)
Anti-Parkinson drugs	309 (7.5)
Drugs for constipation	295 (7.1)
Antihistamines for systemic use	289 (7)

### Panel Attrition

**Figure 2.**
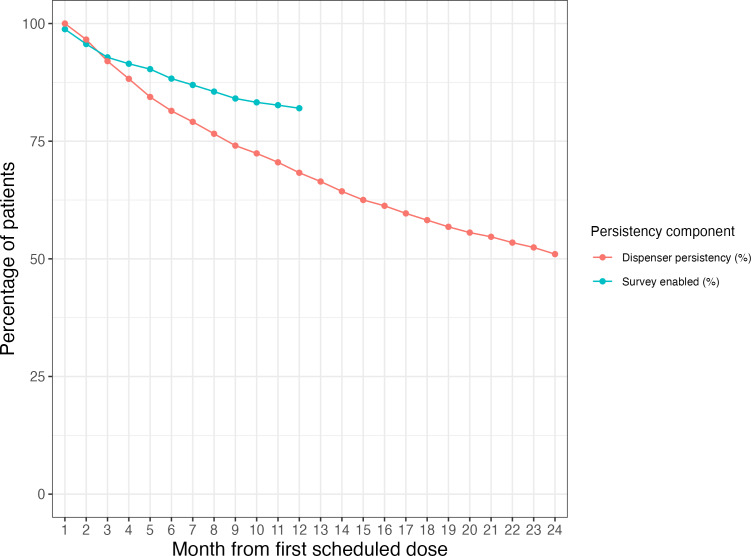
Dispenser persistency and percentage of patients with surveys enabled by month on platform.

From 4138 patients, dispensing persistency was estimated to be 68.3% (95% CI 66.8%‐69.8%) at year 1 and 51% (95% CI 49%‐53%) at year 2. Among the patients who stayed on the dispensing platform past year 1, 82.3% (1508/1832) kept surveys enabled through the 12th month. The rates of question opt-out slowed during the year, as can be seen in Figure 2.

### Survey Response Rates

**Figure 3.**
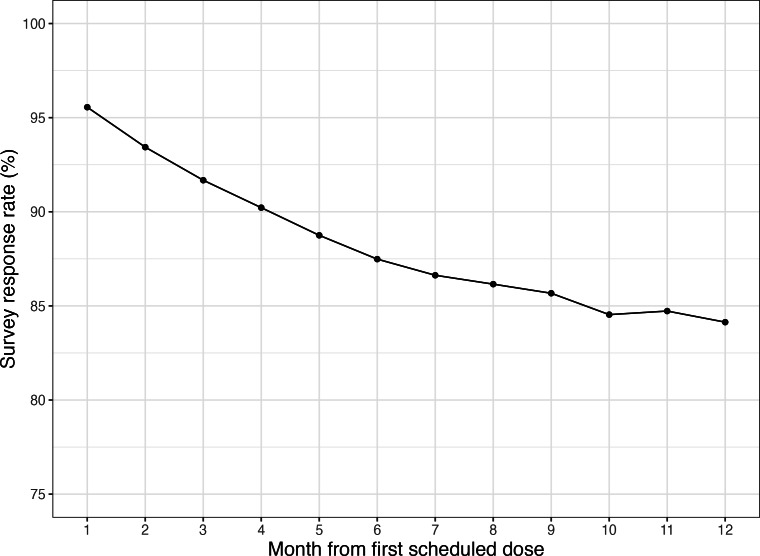
Mean survey response rate (%) for patients with enabled surveys by month on platform.

**Figure 4.**
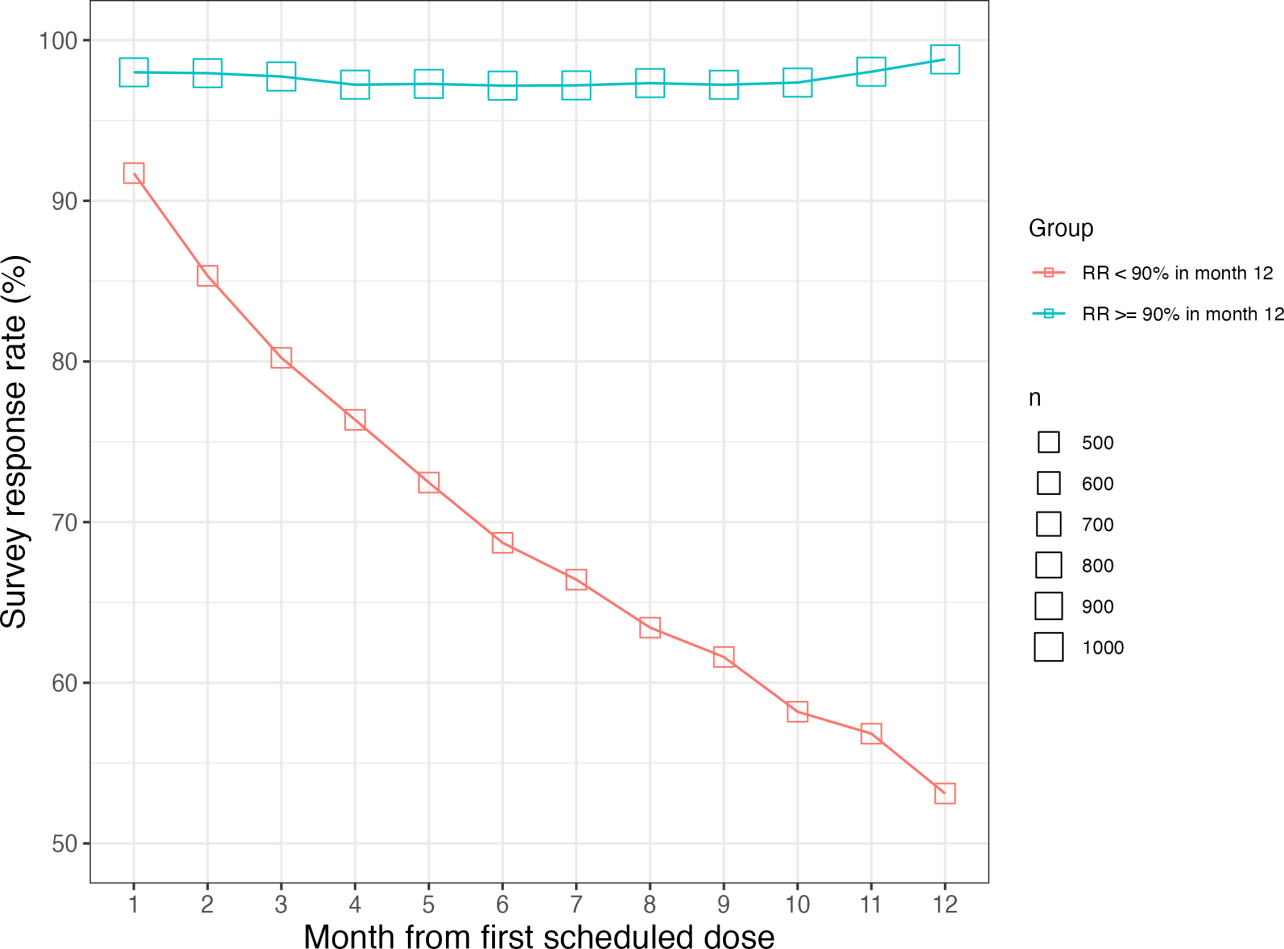
Mean survey RR by month on platform, grouped by RR in the 12th month. RR: response rate.

Among the 1508 patients who kept their surveys enabled through year 1, the mean response rate was 95.6% (SD 11.9%) in the first month and 84.1% (SD 26.4%) in the 12th month, with the rate of decline slowing in the second half of the year (Figure 3). For patients with surveys enabled in the 12th month, 67.9% (1024/1508) had response rates at or above 90% and 32.1% (484/1508) had response rates below 90%. Figure 4 shows the trajectories of both groups, where the high-response group maintained near perfect response rates while the low-response group experienced a substantial decline by the 12th month.

### Reliability

Among 234 patients, the bootstrap estimate of the Pearson correlation between the year 1 and year 2 mean Q_FALL was 0.723 (95% CI 0.630‐0.798), and the estimate of *R*^2^ for the equivalent regression on year 2 versus year 1 means was 0.523 (95% CI 0.397‐0.637).

Mean Q_FALL interval transitions from year 1 to year 2 are shown in Table 3. As hypothesized, self-transitions were the most common, with 59.8% (140/234) of patients starting and staying in [0.5, 1]. These rare fallers in the [0.5, 1.0] interval in year 1 remained in this interval during year 2 in 83.8% (140/167) of cases. For the frequent fallers in the [−1.0, −0.5) interval during year 1, 66.7% (12/18) remained during year 2.

**Table 3.** Mean score range transitions from year 1 to year 2^a^.

Year 1 and year 2 (n=234)		Frequency, n (%)
Frequent fallers [−1.0, −0.5) (n=18)		
	[−1.0, −0.5)	12 (66.7)
	[−0.5, 0.5)	5 (27.8)
	[0.5 to 1.0]	1 (5.6)
Occasional fallers [−0.5, 0.5) (n=49)		
	[−1.0, −0.5)	5 (10.2)
	[−0.5, 0.5)	24 (49)
	[0.5 to 1.0]	20 (48.8)
Rare fallers [0.5, 1.0] (n=167)		
	[−1.0, −0.5)	1 (0.6)
	[−0.5, 0.5)	26 (15.6)
	[0.5 to 1.0]	140 (83.8)

a A mean score of +1.0 would be a “perfect score” of no reported falls, a mean score of −1.0 indicates that all responses indicated that a recent fall had occurred, and scores in between span the interval (−1.0, 1.0). One patient who moved from [−1.0, −0.5) to [0.5, 1.0] had a score of exactly 0.5 (the boundary), with only 6 measurements in year 2.

### Validity

#### Covariates

For 232 patients, the generalized estimating equation model results of Q_FALL on these covariates are shown in Table 4. The coefficients associating biological sex and age to Q_FALL were not significantly different from zero; this was unexpected. However, the coefficient indicating the presence of an SSRI or SNRI medication was negative and highly significant, indicating more falls in the SSRI or SNRI group adjusted for sex and age.

**Table 4.** Generalized estimating equation linear model summary for sex, age, and whether the patient was prescribed a selective serotonin reuptake inhibitor or serotonin-norepinephrine reuptake inhibitor during the observation window.

Coefficient	Estimate	SE	Wald	*P* value
Intercept	0.684	0.127	28.857	<.001^a^
Sex (male)	−0.030	0.069	0.195	.66^b^
Patient age	0.001	0.002	0.092	.76^b^
SSRI^c^ or SNRI^d^	−0.232	0.062	14.092	<.001^b^

a The *P* value corresponding to the hypothesis of the intercept being zero is included by convention but is not a meaningful statistic.

b These *P* values correspond to the 2-sided test of the hypothesis of a zero regression coefficient.

c SSRI: selective serotonin reuptake inhibitor.

d SNRI: serotonin-norepinephrine reuptake inhibitor.

#### Contemporaneous Outcomes From the Spencer

The analysis of correlations between Q_FALL and other contemporaneous spencer questions, based on Kendall τ, is shown in Table 5. Interpretation of correlation coefficients varies, for example, 0.2 may be characterized as either “weak” or “poor,” and 0.3 as “weak,” “moderate,” or “fair” [43], and in the bivariate normal case, a τ value of 0.200 corresponds to a Pearson correlation of 0.309 [44]. While the strength of association between mean Q_FALL and the contemporaneous response outcomes was consistently weak to moderate, *P* values were uniformly small, indicating positive relationships of these questions with the measure of recent falls.

**Table 5.** Contemporaneous association between survey questions administered via spencer.

Question	Patients, n^a^	Average responses per patient, n	Kendall τ	95% bootstrap CI for τ	*P* value^b^
Rate your recent quality of life.	233	14	0.15	0.058-0.244	<.001
How is your emotional health today?	233	54	0.21	0.120-0.293	<.001
How would you rate your physical health today?	164	8	0.18	0.071-0.296	<.001
Rate your ability to perform activities today.	232	35	0.23	0.140-0.316	<.001
Are you able to accomplish what you have planned today?	197	9	0.18	0.083-0.276	<.001
Hospital, ER^c^, or urgent care in the past month?	192	9	0.20	0.096-0.299	<.001

a Number of unique patients who responded to each question at least once and also responded at least 5 times to Q_FALL in year 1 and year 2.

b Derived from Kendall τ test, a nonparametric hypothesis test used to measure the ordinal association between 2 variables.

c ER: emergency room.

## Discussion

### Principal Findings

Although its primary function is dispensing medication, the spencer platform doubled as a web-based longitudinal panel where polychronic patients answered survey questions at high rates and exhibited low panel attrition over years of platform tenure. Measures generated from the responses where stable through time (ie, evidence of reliability) and were associated with other theoretically related variables (ie, evidence of validity). For polychronic patients residing in the US and Canada, the home medication dispenser is a promising source of reliable and valid measures of important health constructs.

As with all survey panels, there was attrition and nonresponse. Panel attrition could be decomposed into attrition from the dispensing platform and survey opt-outs for patients remaining on the dispensing platform. These losses were cumulative. Based on the estimates presented, starting with 100 patients, 68 would still be dispensing via spencer by the end of the first year, with 56 still receiving questions following their dispenses.

In our literature review, persistency was often a serious issue in the context of longitudinal patient studies. For 8 remote digital studies conducted between 2014 and 2019, researchers found that more than half of all participants discontinued their participation within the first week of the study [9]. In a web-based study during the COVID-19 pandemic, of 2734 participants who completed wave 1, only 964 participated in wave 3 [10]. In a study of smartphone app usage to improve oral anticoagulation adherence, a retention rate of 27% at 6 months was reported [11]. Considering these results, keeping more than half of the initial patients actively participating in surveys at the end of the first year represents favorable retention. The rate of new survey opt-outs also decreased substantially through the year, setting up milder losses in year 2.

By the end of first year, the average survey response rate for patients taking surveys on spencer was 84%. While 80% has been considered excellent in the context of primary care research studies [45], multi-item surveys administered at a single point in time are an imperfect benchmark. Ecological momentary assessment, a survey methodology that addresses phenomena as they occur, typically sees compliance rates from 50% to 90% [46]. By either standard, the response rates observed in spencer surveys were good.

We can speculate on why some patients responded to fewer spencer questions over time than others. Survey fatigue is a well-known phenomenon that occurs when respondents become weary of repeated survey tasks [47], and although surveys administered via spencer are brief, they are frequent. In addition to fatigue, some patients may not have been aware of how the questions were being used to monitor their well-being. Developing interventions to improve survey response rates is a topic for future research.

Noncoverage in web-based surveys, often defined as lack of access to the web, is thought to be a more serious problem than nonresponse, which is an unwillingness to participate [48]. Since every participant has a connection to the web through the device itself (the spencer units have both cell and Wi-Fi connections), there is no noncoverage in the sense of lack of web access, although machines do go offline for varying durations.

With sufficiently high panel persistency and response rates, attention focuses on the quality of the data that are generated. We showed that a measure about recent falls generated from a spencer question exhibited temporal stability, a form of reliability. The measure showed expected associations with most theoretically related variables. One exception was the demographic factors of age and biological sex, which failed to achieve significance in a regression where medication use was significant (*P*<.001). While additional data may reveal the expected relationships, we surmise that in a polychronic population taking many medications, demographic factors may be weaker predictors of falling than in the general population.

### Limitations

First, since the patients studied in this paper were polychronic patients enrolled in a care management program and residing in the United States and Canada, inferences to other populations may not be warranted.

Second, our validity analyses were limited to data collected entirely within the spencer ecosystem. While correlations between spencer survey responses suggest meaningful patterns, these questions, although having face validity, lack validation against established instruments. The observed correlations might partly reflect the consistent presentation format on the device, where responses are always ordered from most to least positive sentiment. Our validity arguments would be substantially stronger with independent measurements, particularly comparisons between spencer responses and validated traditional instruments measuring the same constructs.

Third, this study did not consider sensitivity to change, which is important in the context of RWE because it allows researchers and clinicians to detect change resulting from a minimal intervention [49]. While we focused on reliability by treating fall risk as a stable construct, and although fall risk is sufficiently stable to support our reliability analysis, treating it as static was a limitation of this research. Future research could explore methods for estimating changing states from longitudinal survey data, building on established approaches in the literature [50,51].

### Conclusions

Administering longitudinal surveys via spencer, a smart medication dispenser, effectively generated high-quality RWD from patients in their homes. Patients persisted on the platform for years and maintained high response rates. A measure derived from longitudinal surveys assessing fall risk demonstrated both reliability and validity. The performance of spencer as a longitudinal survey platform offers a promising alternative at a time when web-based survey data quality is deteriorating.

Because medication dispensing is a fundamental component of the survey-generating mechanism, RWD from the spencer platform offers an ideal opportunity to study medication effectiveness and health outcomes, providing evidence to support new drug indications and demonstrate relationships between health outcomes and economic factors.

## Abbreviations

IRB: institutional review board
MTurk: Amazon Mechanical Turk
PRO: patient-reported outcome
Q_FALL: question about recent falls asked on spencer
RWD: real-world data
RWE: real-world evidence
SNRI: serotonin-norepinephrine reuptake inhibitor
SSRI: selective serotonin reuptake inhibitor
